# Negative Selection Assay Based on Stimulation of T Cell Receptor Transgenic Thymocytes with Peptide-MHC Tetramers

**DOI:** 10.1371/journal.pone.0043191

**Published:** 2012-08-10

**Authors:** Vasily Rybakin, Nicholas R. J. Gascoigne

**Affiliations:** Department of Immunology and Microbial Sciences, The Scripps Research Institute, La Jolla, California, United States of America; Oklahoma Medical Research Foundation, United States of America

## Abstract

Thymocyte negative selection is a requirement for the development of self tolerance. Although it is possible to assay the induction of cell death in thymocytes in vitro using antibody cross-linking, this stimulus is much stronger than the normal range of T cell receptor ligands that could be encountered during normal development. Signaling in thymocytes is finely balanced between positive and negative selection stimuli, where a negative selecting ligand can be only marginally higher affinity than a positive selecting ligand. We have therefore developed an assay for the induction of negative selection that can distinguish such cases, and that is amenable to high-throughput analysis. The assay is based on the induction of activated caspase 3 in thymocytes expressing a defined T cell receptor, after stimulation with MHC-peptide tetramers in vitro for 24 hours or less.

## Introduction

Each conventional mature T cell expresses one variety of T cell receptor (TCR) on its surface and is capable of precisely recognizing and responding to an extremely low dose of an appropriate agonist peptide presented on major histocompatibility complex (peptide-MHC, pMHC) by an antigen presenting cell. The repertoire of valid receptors is crucial to ascertaining that the adaptive immune system can recognize non-self or modified self threats while specifically excluding autoreactivity. Such selectivity is achieved during thymic selection of T cells and involves two fundamental phenomena. Thymocytes expressing functional TCR on their surface successfully pass the positive selection test in the thymic cortex whereas cells failing to achieve survival signal via TCR-pMHC interaction are eliminated by “death by neglect” [1]–[3]. Surviving cells then proceed to the thymic medulla where expression of Aire drives presentation of a wide range of non-thymic autoantigens on medullary thymic epithelial cells [4]. During negative selection, strong interactions with such antigens presented by medullary thymic epithelial cells, or cross-presented on dendritic cells, result in the activation-induced cell death and elimination of potentially autoreactive specificities. Thymocytes surviving both positive and negative selection become mature T cells and migrate to the periphery [1], [2].

Ex vivo simulation of negative selection has been extensively used to study signaling events downstream of TCR stimulation in immature thymocytes. The most widely used model is the titration of crosslinking CD3 antibody [5]–[7] in the presence of CD28 costimulation. In such assays, it is assumed that minimal concentrations of anti-CD3 mimic positive selection because the combined signaling input from all expressed TCRs is low, while at high concentrations high occupancy of TCR leads to strong signals characteristic of negative selection. While in terms of very gross selection outcomes TCR crosslinking may mimic the absolutely lowest and highest selection pressures (homeopathic TCR antibody concentrations will not yield any signal while effective crosslinking will lead to cell death), we believe that it does not represent an appropriate model for the analysis of the fine thresholds between life and death signaling during negative selection. Stimulation of TCR transgenic thymocytes with pMHC ligands of marginally different potency can result in vastly different selection outcomes [8]–[10].

The CD4/8 dulling assay [11]–[13], while representing a feasible quantitative approach for studying both positive and negative selection ex vivo, does not directly reflect developmental fate of preselection thymocytes following stimulation. Other ex vivo models of thymic selection include incubation of TCR-transgenic thymocytes with antigen presenting cells loaded with relevant peptides [14]. This stimulation is much more physiological than TCR crosslinking, but the presence of another cell type makes biochemical and genetic analysis of stimulated thymocytes significantly more complicated. In fetal thymocyte organ culture assays, whole embryonic thymic lobes are cultured ex vivo in the presence of peptides of interest [15], [16]. While FTOC accurately mimics thymic selection and is extremely useful in the analysis of intrinsic developmental properties of immature thymocytes under a variety of conditions, the presence of multiple cell types makes characterization of developing thymocytes by methods other than flow cytometry extremely difficult.

**Figure 1:**
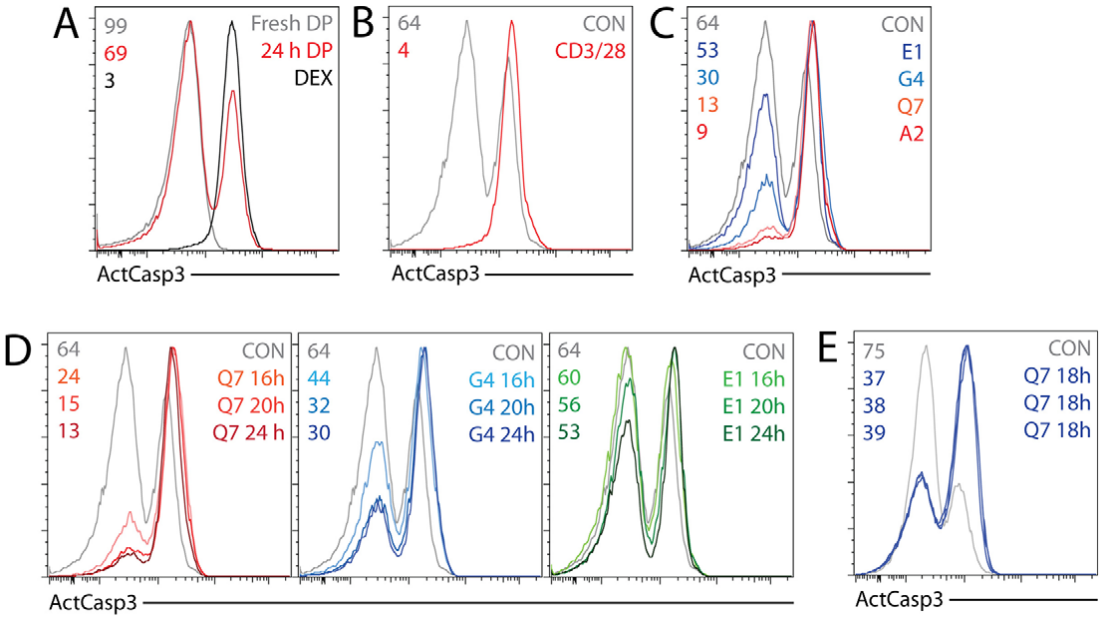
ActCasp3 staining reflects selection outcomes in response to tetramers with different affinities for TCR. (A) Assay validation: Freshly isolated B6 thymocytes (gray) were compared with cells cultured for 24 h in the presence (black) or absence (red) of 5 µM dexamethasone. OT-I *Tap1* ^−/−^ thymocytes were stained for CD4 and CD8, fixed, and stained for ActCasp3. Histograms represent ActCasp3 staining (X axis) in the CD4^+^8^+^ DP gate. Numbers in this and all following FACS plots represent percentage of DP thymocytes in ActCasp3^−^ gate. (B) ActCasp3 staining in the DP gate with and without TCR crosslinking with magnetic beads coated with anti-CD3 and anti-CD28 antibodies for 24 h. (C) ActCasp3 staining in the DP gate with and without stimulation with A2, Q7, G4, and E1 H-2K^b^ tetramers for 24 h; (D) ActCasp3 staining in the DP gate with and without stimulation with Q7, G4, and E1 H-2K^b^ tetramers for 16–24 h.

We propose a new ex vivo model for analysis of negative selection based on stimulation of TCR transgenic thymocytes with pMHC tetramers with subsequent analysis of TCR signaling-induced apoptosis by activated caspase 3 staining. This fast, straightforward and quantitative method can be used to analyze signaling pathways contributing to selection, gene expression downstream of a variety of signals, and to screen drug libraries with the aim of identifying new compounds affecting early T cell fate. Active caspase 3 staining was selected as marker of apoptosis induction because it represents a comparatively early way of detecting apoptosis [17] and allows for high throughput analysis of large numbers of samples by high throughput flow cytometry [18]. Thymocyte apoptosis induced by TCR crosslinking has been shown to be caspase 3 mediated [19], [20], and activation of caspase 3 is a hallmark of an irreversible commitment to apoptosis [21]. However, while undoubtedly occurring during apoptosis after negative selection signaling, caspase 3 activation may not be strictly required for negative selection. Elimination of thymocytes can occur in the absence of caspase 3 in H-Y and P14 TCR-transgenic systems, likely due to compensation by other caspases and/or caspase-independent pathways [22].

**Figure 2:**
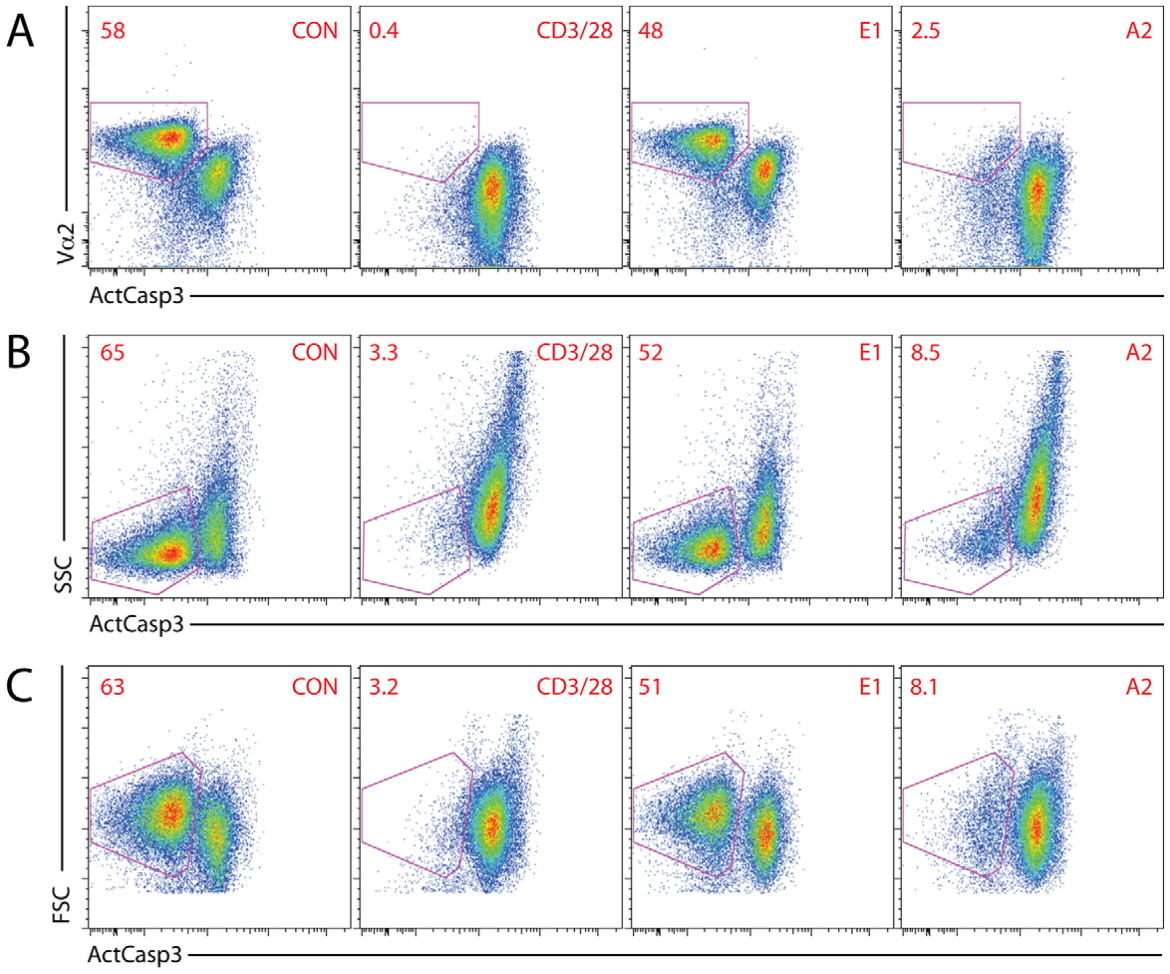
Alternative gating strategies. (A) ActCasp3 (Y axis) and Vα2 staining (X axis) in the DP gate with and without stimulation with anti-CD3/28 beads or E1 and A2 H-2K^b^ tetramers for 24 h. (B) ActCasp3 (Y axis) and side scatter signal (X axis) in the DP gate with and without stimulation with anti-CD3/28 beads or E1 and A2 tetramers for 24 h. (C) ActCasp3 (Y axis) and forward scatter signal (X axis) in the DP gate with and without stimulation with anti-CD3/28 beads or E1 and A2 tetramers for 24 h.

## Materials and Methods

### Ethics statement

Animal use was approved by the Institutional animal care and use committee of TSRI (protocol #06-0340).

*Mice.* C57BL/6 (B6) and OT-I *Tap1* ^−/−^ mice were bred and maintained at the TSRI animal facility.

### Cells and stimulation

Thymocytes from 4 week old mice were adjusted to 2.5x10^6^/mL with phenol red free RPMI containing 10% fetal calf serum, 100 U/ml penicillin, 10 µg/ml streptomycin, 292 µg/ml glutamine, 50 µM β-mercaptoethanol and 1 µg/ml anti-CD28 antibody (LEAF purified, clone 37.51, Biolegend) and plated in 96-well U-bottom plates at 200 µl/well. For tetramer stimulation, PE labeled H-2K^b^ tetramers with various peptides (NIH Tetramer Core Facility) were added at 2 µl per well. Peptides used were: A2 (SAINFEKL), Q7 (SIINFEQL), G4 (SIIGFEKL), E1 (EIINFEKL). For TCR crosslinking, magnetic beads coated with anti-CD3 and anti-CD28 antibodies (Invitrogen) were added at 0.5x10^6^ beads/well. For positive control of apoptosis induction, cells were incubated with 5 µM dexamethasone. Cysteine protease inhibitor Z-VAD (OMe)-FMK (EMD Millipore) was used at 200 µM. Plates were incubated at 37°C and 5% CO2 for 15–24 h. Where indicated, cells were stained with 5 µM Proliferation Dye 670 (eBioscience) in PBS for 10 min in the dark and destained with 10% FBS for 10 min prior to plating.

**Figure 3:**
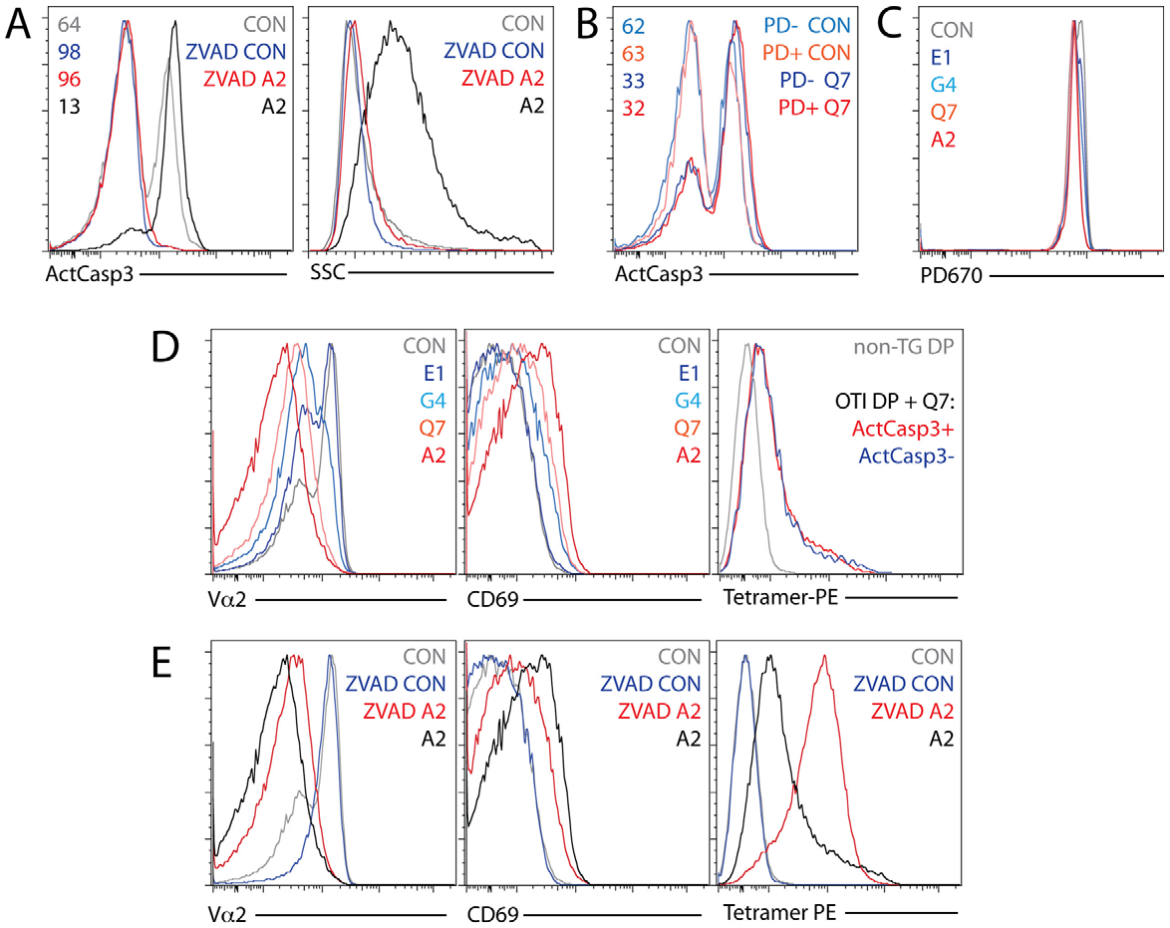
Assay specificity and possible additional analyses. (A) ActCasp3 staining (left) and side scatter signal (right) in the DP gate with and without A2 tetramer stimulation for 24 h in the presence or absence of caspase inhibitor Z-VAD (OMe)-FMK. (B) Simultaneous stimulation of PD670-labeled and unlabeled cells in the same well with K^b^-Q7 tetramer for 24 h. ActCasp3 staining in the PD670 positive and negative DP gate with and without stimulation with tetramer for 24 h. (C) PD670 signal (X axis) in the DP gate with and without stimulation with A2, Q7, G4, and E1 tetramers for 24 h. (D) Vα2 (left) and CD69 (middle) staining in the DP gate with and without stimulation with A2, Q7, G4, and E1 tetramers for 24 h. Tetramer-PE signal (right) in ActCasp3^+^ and ActCasp3^−^ DP gate compared with that of non-treated B6 DP thymocytes. (E) Vα2 (left), CD69 (middle) and tetramer-PE (right) signals in the DP gate with and without stimulation with K^b^-A2 tetramer for 24 h in the presence or absence of caspase inhibitor Z-VAD (OMe)-FMK.

### Staining and analysis

After stimulation, cells were placed on ice and stained with surface antibodies (CD4-eFluor450 clone GK1.5, CD8-PE-Cy7 clone 53–6.7, both eBioscience, and Vα2-PerCP-Cy5.5 clone B20.1, BD). Fixation, permeabilization, and staining for activated caspase 3 were according to manufacturer's instructions (Cell Technology cat. # FAB200-2). Cells were analyzed on BD LSRII flow cytometer. For quantitative analysis of apoptosis, percentage of active caspase 3 negative cells was calculated within the CD4^+^CD8^+^ double positive (DP) gate.

## Results and Discussion

Intracellular staining for activated caspase 3 (ActCasp3) with subsequent analysis by flow cytometry has been extensively used for quantitative analysis of apoptosis in a variety of model systems [23]–[26]. Here, we used ActCasp3 stain as readout for thymocyte negative selection. Freshly isolated CD4^+^8^+^ (“double positive”: DP) thymocytes from B6 mice did not show significant ActCasp3 signal since cells dying during selection in vivo are rapidly eliminated by macrophages [27], [28]. After 24 hex vivo incubation without stimulation, 25–35% of DP thymocytes were apoptotic and 65–75% of cells survived, while addition of 5 µM dexamethasone (positive control) decreased the percentage of non-apoptotic cells to 1–3% (**Fig.** **1A**).

As expected, TCR crosslinking for 24 h elicited a signal strong enough to eliminate a similar percentage of cells to dexamethasone treatment (**Fig.** **1B**). Stimulation of OT-I *Tap1* ^−/−^ thymocytes for 24 h with a high affinity agonist tetramer (K^b^-A2) for 24 h yielded near complete elimination of thymocytes and was comparable with TCR crosslinking, while decreasing potency tetramers (A2>Q7>G4>E1) resulted in gradual increase in cell survival (**Fig.** **1C**). (Binding kinetics for the OT-I TCR to the MHCp as either monomers or tetramers, and their ligand potencies, were determined previously [8]–[10], [29]). We found no difference between tetramer-only and tetramer plus anti-CD28-stimulated samples in these experiments (data not shown). Shorter incubations resulted in lower percentages of apoptotic cells, as expected (**Fig.** **1D**). Higher potency tetramers (K^b^-A2, K^b^-Q7, K^b^-G4) led to rapid induction of cell death plateauing at 20h, whereas weak tetramers (K^b^-E1) did not induce apoptosis at 16 h incubation time, but gradually increased the percentage of cells positive for ActCasp3 at 20 h and 24 h. Investigators need to empirically determine the correct time frame for analysis after stimulation of interest. In particular, differences in comparative kinetics and efficiencies of apoptosis induction between control and experimental samples may manifest themselves only within a very specific time window.

We tested the reproducibility of the ActCasp3 staining after tetramer stimulation by performing stimulation in technical triplicates. Sample to sample variability was on average between 2–4% (**Fig.** **1E**).

Since under certain circumstances the analysis of cell subpopulations by binary gating on histograms can be prone to errors [30], we investigated additional stains to increase the accuracy of gating. We noticed that 2D gating based on ActCasp3 and TCR (in this case using anti-Vα2) resulted in clear separation between ActCasp3^+^, TCR^intermediate^ (apoptotic) and ActCasp3^−^ TCR^high^ (live) cells (**Fig.** **2A**). Simpler but still effective 2D gating can be based on active caspase 3 staining and side or forward scatter (**Fig.** **2B, C**). Apoptotic, ActCasp3^+^ cells display higher side scatter signal and lower forward scatter signal than live cells.

For assay validation, we analyzed cells incubated with cell-permeable irreversible pan-caspase inhibitor Z-VAD (OMe)-FMK at 200 µM in the presence or absence of tetramer stimulation. As shown in **Fig.** **3A**, caspase inhibition completely eliminated ActCasp3 staining in both tetramer-stimulated and non-stimulated samples. K^b^-A2 tetramer stimulation in the presence of inhibitor did not result in the increased side scatter profile characteristic of apoptotic cells (compare to **Fig.** **2B**), which is in agreement with the finding that inhibition of caspase activity in general, and caspase 3 in particular, may be sufficient to prevent apoptosis [31], [32].

We used thymocytes loaded with Proliferation Dye 670 (PD670) to test the possibility of simultaneous analysis of mixed samples in the same well. Such analysis is potentially capable of reducing false sample-to-sample variability due to handling artifacts such as pipetting errors [33]. We combined PD670 stained and unstained thymocytes at 1∶1 ratio, stimulated with K^b^-Q7 tetramer or left unstimulated for 24 h, and analyzed for ActCasp3 (**Fig.** **3B**). The procedure of staining with PD670 did not affect cell viability, resulting in comparable ActCasp3 staining in PD670-positive and negative gates. The proposed assay has therefore good potential for analysis of two or more distinct populations in the same well (e.g. control versus knockout or control versus drug treated sample). Neither untreated nor tetramer-stimulated cells proliferated within 24 h of incubation, as demonstrated by the lack of PD670 dilution (**Fig.** **3C**).

More detailed analysis of tetramer-stimulated cells revealed that higher potency ligands yielded stronger TCR downregulation and higher expression of CD69, indicative of stronger TCR signaling (**Fig.** **3D**). Importantly, distinct populations downregulating TCR or failing to do so, did not arise due to the lack of binding of tetramer to the latter as gating on ActCasp3^+^ and ActCasp3^−^ cells showed comparable tetramer signal. Stimulation in the presence of caspase inhibitor Z-VAD (OMe)-FMK resulted in slightly decreased TCR downregulation and CD69 expression but very strong tetramer signal (**Fig.** **3E**). Higher tetramer accumulation may be reflective of a requirement for cysteine protease activity for the dissociation and degradation of internalized TCR-pMHC complexes.

To summarize, we propose a new method for quantitatively characterizing negative selection. Instead of titrating anti-TCR antibodies, we utilize tetramers with different affinities to a transgenic TCR. As expected, increasing pMHC tetramer potency led to an increased percentage of cells inducing apoptosis as indicated by ActCasp3 staining. We expect that this method will be useful for more accurate investigation of cell signaling mechanisms leading to the discrimination between weak, intermediate, and strong signals during thymocyte selection, and that it will be useful for screening of molecules that can change selection outcomes. A few limitations of the proposed system need to be considered prior to setting up experiments. (1) If the experimental design requires comparison of selection thresholds in wild type and mutant mouse thymocytes in response to the same signal, then mutant mice will need to be crossed to the OT-I *Tap1* ^−/−^ (or similar) background, which may be a lengthy process. (2) When adapting the method to high throughput screening (HTS), it is important to consider that multiple fixation, permeabilization, staining, and wash steps may lead to significant loss of thymocytes from suspension. Feasibility of attaching thymocytes to poly-lysine or other substrates needs to be experimentally evaluated. In addition, substitution of intracellular staining with anti-activated caspase antibody by fluorescently labeled caspase reporters, or use of another apoptosis marker such as annexin V, may simplify the protocol for HTS. (3) While the system may be supplemented by additional biosensors to track signaling events preceding caspase activation, e.g. phosphorylation of signaling molecules in the TCR cascade, it will likely be difficult to empirically determine stimulation time points short enough to report on TCR signaling yet long enough for the induction of apoptosis. Additionally, we anticipate that shorter incubation times will result in increased variability of results.
